# Blood genomic profiles of exposures to Venezuelan equine encephalitis in Cynomolgus macaques (*Macaca fascicularis*)

**DOI:** 10.1186/1743-422X-4-82

**Published:** 2007-08-29

**Authors:** Rasha Hammamieh, Mohsen Barmada, George Ludwig, Sheila Peel, Nick Koterski, Marti Jett

**Affiliations:** 1 Division of Pathology, Walter Reed Army Institute of Research, Silver Spring, MD, USA; 2 Office of the Principal Assistant for Research and Technology, United States Army Medical Research and Materiel Command, Frederick, MD, USA; 3 Division of Retrovirology, Walter Reed Army Institute of Research, Rockville, MD, USA; 4 Division of Virology, United States Army Medical Research and Materiel Command, Frederick, MD, USA

## Abstract

**Background:**

Lymphocytes provide invaluable whistle blowers of changes due to infections. We use the information registered by these cells using their mRNAs as they encounter the pathogen to develop patterns of expression that correspond to that specific pathogen.

Venezuelan equine encephalitis (VEE) is a mosquito-borne viral disease characterized by fever and one or more of the following: severe headache, back pain, myalgias, prostration, chills, nausea, vomiting, weakness and other flu-like symptoms.

Screening for host mRNA obtained from blood samples after exposure to VEEV may provide the means for early detection of surrogate markers of the impending illness and provide appropriate strategies for treatment.

**Results:**

We have been carrying out gene expression analysis of PBMC exposed to VEEV to extract signatures and diagnostic markers of early exposure to be used in non invasive blood analysis methods.

In this study, we used high throughput gene expression analysis to identify markers of early and late exposures to VEEV in vivo in Cynomolgus macaques (*Macaca fascicularis*). We carried out cDNA microarrays and real time PCR on blood samples obtained from the NHP model resulting in a panel of host genes that are altered in response to VEEV.

**Conclusion:**

Screening for host mRNA obtained from blood samples after exposure to VEEV may provide the means for early detection of surrogate markers of the impending illness and provide appropriate strategies for treatment.

## Background

A reliable and rapid diagnosis of viral infections has long been a major concern for clinicians due to the fact that most of viral diseases exhibit flu-like symptoms early on in the course of the illness and effective treatment often requires intervention early in the course of disease. Detection of exposure to viral pathogens has relied on ever more sensitive methods for pathogen identification. Assessing exposure to a pathogen well in advance of onset of illness or at various stages post-exposure would be invaluable to clinicians. To counter the threat of biological attack and emerging diseases, it is critical to develop the capability to distinguish accurately between a common infection such as seasonal influenza and exposure to a biological weapon or newly emerged or newly introduced pathogen.

Lymphocytes, in their role as purveyors of humoral immunity, may serve as invaluable indicators of the changes that occur in response to particular infectious processes. By monitoring their evolving pattern of mRNA production as they encounter a pathogen, we may be able to define patterns of expression that correspond to that specific pathogen. Venezuelan equine encephalitis (VEE) is a mosquito-borne viral disease caused by an enveloped single-stranded RNA virus of the family Togaviridae, genus Alphavirus [1]. Members of the virus complex that cause VEE are endemic to different parts of South America, Trinidad, Central America, Mexico, and Florida. Disease caused by members of the VEE virus (VEEV) complex is usually characterized by fever and one or more of the following: severe headache, back pain, myalgias, prostration, chills, nausea, vomiting, and weakness and it may rarely progress to encephalitis [2-4].

The aerosol form of VEEV is highly infectious, making VEEV a potential biowarfare agent. If this virus was deployed efficiently, it could incapacitate significant numbers of people for a week or more and cause untold psychological stress to millions [5,6]. Like many other viruses, VEEV is potentially susceptible to genetic manipulation which could compound its virulence or render it invisible to sequence-specific diagnostic identification [3].

Diagnosis of VEE has traditionally relied on viral isolation from acute phase serum or spinal fluid, IgG levels in paired serum samples, or on detection of VEEV-specific IgM in serum or the cerebrospinal fluid[7]. Recently, PCR based assays have been developed and employed in many testing laboratories for detection of VEEV infections.

The objective of the current study is to examine the early cellular and molecular changes induced in peripheral blood mononuclear cells (PBMC) of a non-human primate model in response to exposures to VEEV, which likely mirror the initial stages of infection in the human host.

We studied gene expression profiling in response to VEEV infection in cynomolgus macaques (*Macaca fascicularis*) that were used as part of a larger study carried out by the Department of Defense to assess the host pathological responses to VEEV.

In this report we identify biomarkers for exposures to VEEV obtained from blood samples. Screening for host mRNA obtained from PBMCs after exposure to VEEV may provide the means for early detection of surrogate markers of the impending illness.

The ability to identify specific gene patterns early on can provide appropriate strategies for prevention or treatment that would lead to amelioration of the disease progression.

Note: microarray data have been submitted to the Gene Expression Omnibus (GEO) and can be searched using the Platform ID: GPL5486.

## Results

### Microarray analysis of VEEV infected vs. uninfected NHPs

Inter-chip and intra-chip data normalizations were computed using GeneSpring (Agilent, CA), as described in the methods section. One-way ANOVA with a P-value < 0.05 identified 1378 genes of interest; listing the most differentially expressed genes between the control and VEEV exposed NHPs.

**Figure 1:**
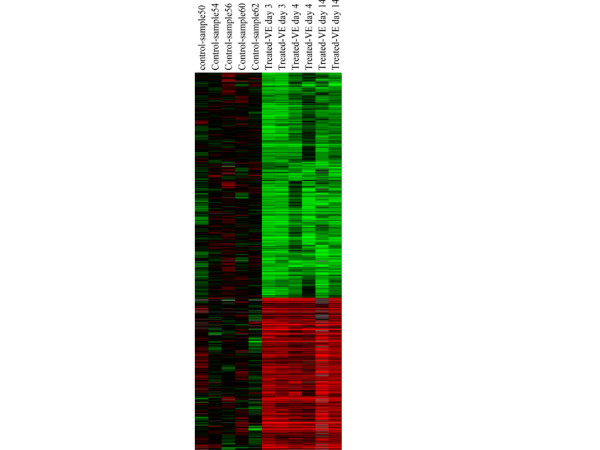
Cluster view of gene expression profiles showing altered regulation of genes induced by VEEV in PBMC. Blood was collected at various time point post exposure to VEEV. RNA was isolated, hybridized to human cDNA arrays, scanned and data analyzed using Gene Spring. Red shows up regulated and green represents down regulated genes compared to control unexposed animals. Cluster analysis was performed using the Hierarchical cluster and Tree view.

**Figure 2:**
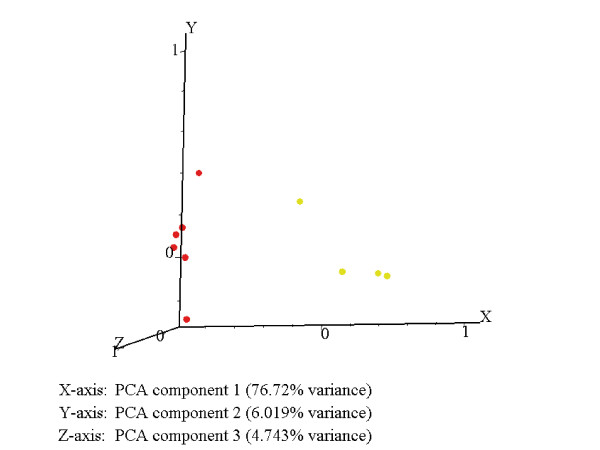
Principal component analysis of gene expression profiles in VEEV infected compared to control animals. Although the animals were clinically reported asymptomatic, the VEEV treated and control samples cluster far from each other along PCA1 axis.

Figure 1 is a cluster view of genes differentially expressed between the control and VEEV exposed NHPs. We carried out PCA on the control and treated samples. Figure 2 shows that samples from NHPs exposed to VEEV were clustered together and maintained a significant distance from the control group along first principal component axis (x-axis: PCA1), which, incidentally, represents the highest variance between the two groups.

### Confirmation of gene expression changes by Real-Time PCR analysis

**Figure 3:**
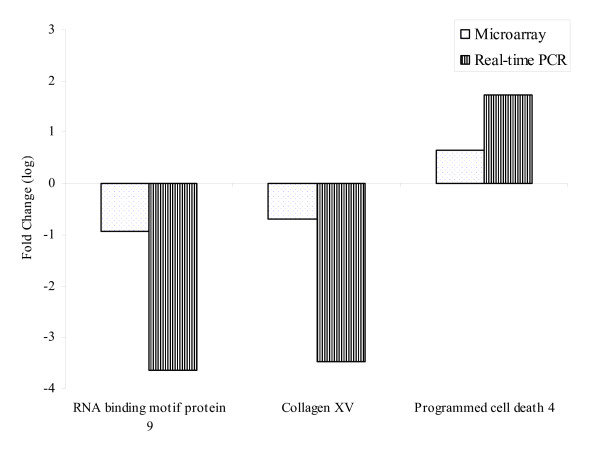
A comparative analysis of four selected genes using array analysis and Real-time PCR. RNA binding motif protein 9 and collagen, type XV, were down regulated in VEEV infected animals while programmed cell death 4 was down regulated.

Four genes were selected for real-time polymerase chain reaction (PCR). They are RNA binding motif protein 9 (AA451903), collagen, type XV, programmed cell death 4 (N71003), and the house keeping gene, GAPDH. Figure 3 illustrates that the real-time PCR expression profiles for the selected genes are well correlated with the corresponding microarray results.

### Data mining of genes differentially expressed between control and VEEV exposed NHPs

We used GeneSpring 7.1 and FATIGO+ [8] to functionally classify genes and identify pathways that were regulated by the VEEV in the blood of exposed NHPs.

**Figure 4:**
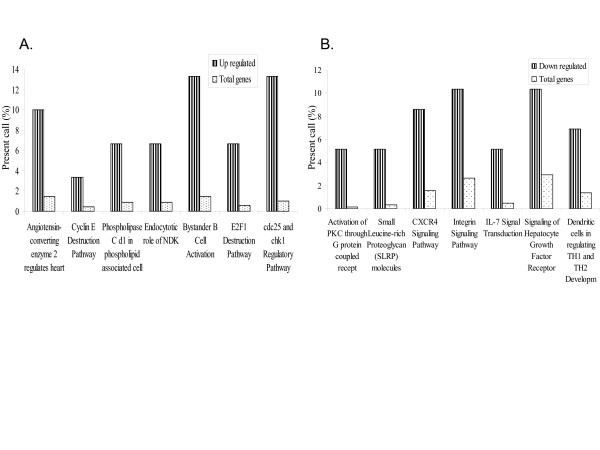
Ontological analysis of the genes that were up (a) or down (b) regulated by VEEV in PBMC. RNA samples were isolated and hybridized on the cDNA microarray slides as detailed in materials and methods. Images were analyzed using GenePix 4.0 and data were analyzed using GeneSpring 7.0. Data were then analyzed using FATIGO^+ ^to identify functional classes regulated by the virus. We calculated the percentage of each ontological class found in the list of genes regulated by VEEV and compared it to the percentage of found in the total gene list of the cDNA array.

Using FATIGO^+ ^and GeneCite [9] we carried out a detailed pathway analysis using the Biocarta pathways [10]. Figure 4 shows pathways differentially regulated by VEEV in the blood samples.

Gene ontological classification, using FATIGO^+ ^and GeneCite [9], of genes regulated by the VEEV in the blood suggested that genes related to immune defense, transcription factors, cell adhesion, cell growth, apoptosis and signal transduction were regulated by the virus.

Table 2 represents the functional classification of some of the genes of interest.

**Table 2:** Functional classification of some of the genes of interest.

**Gene ID**		**Fold Change**
**Toll-Like Receptors**		

AF051151	toll-like receptor 5	1.46 ± 0.50
AF177765	toll-like receptor 4	0.67 ± 0.27
AL050262	toll-like receptor 1	0.41 ± 0.13
AL570789	toll-like receptor 3	1.47 ± 0.48
NM_003264	toll-like receptor 2	0.50 ± 0.19

**Cytokines**		

NM_001558	interleukin 10 receptor, alpha	0.18 ± 0.09
S36219	prostaglandin-endoperoxide synthase 1	0.30 ± 0.06
AV707896	Small inducible cytokine subfamily E, member 1	0.33 ± 0.11
D87931	Rho-associated, protein kinase 2	0.40 ± 0.13
NM_003809	tumor necrosis factor (ligand) superfamily,	0.42 ± 0.25
NM_005211	colony stimulating factor 1 receptor	0.46 ± 0.18
NM_001380	dedicator of cytokinesis 1	0.47 ± 0.24
NM_004120	guanylate binding protein 2, interferon-inducible	1.91 ± 0.29
NM_002988	chemokine (C-C motif) ligand 18	2.23 ± 0.81
NM_002186	interleukin 9 receptor	3.17 ± 2.37
AB006967	suppressor of cytokine signaling 3	3.60 ± 2.87
NM_005408	chemokine (C-C motif) ligand 13	6.00 ± 3.46

**Neuronal related genes**		

NM_005045	reelin	0.32 ± 0.12
NM_006334	olfactomedin 1	0.34 ± 0.17
AF165124	gamma-aminobutyric acid (GABA) A receptor,	0.35 ± 0.11
AU134339	Ceroid-lipofuscinosis, neuronal 3, juvenile	0.37 ± 0.22
NM_000727	calcium channel, voltage-dependent, gamma	0.43 ± 0.18
X57548	cadherin 2, type 1, N-cadherin (neuronal)	0.44 ± 0.05
NM_000742	cholinergic receptor, nicotinic, alpha 2 (neuronal)	0.44 ± 0.18
NM_005612	RE1-silencing transcription factor	0.49 ± 0.15
NM_002738	protein kinase C, beta 1	0.49 ± 0.30
BG169625	Enolase 2 (gamma, neuronal)	0.50 ± 0.20
NM_001830	chloride channel 4	0.55 ± 0.28
M34064	cadherin 2, type 1, N-cadherin (neuronal)	0.58 ± 0.38
NM_002522	neuronal pentraxin I	0.61 ± 0.44
AF166003	potassium voltage-gated channel, member 1	1.95 ± 0.68
NM_004061	cadherin 12, type 2 (N-cadherin 2)	1.98 ± 0.82
AA975079	Ankyrin 2, neuronal	2.22 ± 0.57
NM_000620	nitric oxide synthase 1 (neuronal)	2.27 ± 0.38
AI986443	Similar to neuronal pentraxin receptor isoform 2	2.60 ± 0.40
AK001991	leucine rich repeat neuronal 3	2.80 ± 1.35
AF169693	protocadherin 20	3.02 ± 1.50
NM_014211	gamma-aminobutyric acid (GABA) A receptor, pi	3.17 ± 2.99
X90846	mitogen-activated protein kinase kinase kinase 10	6.00 ± 1.85
AA243675	Solute carrier family 1	7.37 ± 2.78
AI912373	Neuronal guanine nucleotide exchange factor	29.3 ± 7.91

**Caspase pathway**		

NM_001223	caspase 1	0.26 ± 0.16
NM_004131	granzyme B	1.68 ± 0.47
AU125557	Caspase 3	2.09 ± 0.88
H22169	Lamin A/C	3.62 ± 0.80
NM_001230	caspase 10	4.43 ± 1.33
AA041298	Caspase 4	28.3 ± 5.56

**Induction of Apoptosis**		

AF181850	inhibitor of growth family, member 1	2.09 ± 0.40
AI591151	parathyroid hormone-like hormone	21.6 ± 7.25
L26165	cyclin-dependent kinase inhibitor 1A	2.11 ± 0.84
NM_000546	tumor protein p53	2.37 ± 1.05
NM_001230	caspase 10	4.43 ± 1.33
NM_002048	growth arrest-specific 1	4.14 ± 0.80
NM_003123	sialophorin (leukosialin, CD43)	3.01 ± 1.10
X90846	mitogen-activated protein kinase kinase kinase 10	6.00 ± 1.85

**Integrins**		

BG032225	integrin beta 1 binding protein 1	0.63 ± 0.06
N95414	Integrin, alpha 2	0.39 ± 0.23
NM_000885	integrin, alpha 4	0.50 ± 0.21
NM_000887	integrin, alpha X	0.27 ± 0.17
AW513695	Integrin, beta 1	0.46 ± 0.18
AL581999	Integrin, beta 7	0.31 ± 0.07
AA569711	Integrin, beta 8	0.65 ± 0.15

**Immune Response**		

M12824	CD8a molecule	0.20 ± 0.12
NM_002647	phosphoinositide-3-kinase, class 3	0.23 ± 0.11
NM_003998	nuclear factor of kappa light polypeptide	0.32 ± 0.11
NM_000569	Fc fragment of IgG, low affinity IIIa, receptor	0.34 ± 0.11
NM_001734	complement component 1, s subcomponent	0.34 ± 0.18
AF077196	regulatory factor X-associated ankyrin-containing	0.36 ± 0.14
AL050262	toll-like receptor 1	0.41 ± 0.13
X14831	carcinoembryonic antigen-related cell adhesion	0.42 ± 0.20
S82807	thyroid stimulating hormone receptor	2.17 ± 1.22
L13210	lectin, galactoside-binding protein	2.32 ± 0.42
AF004231	leukocyte immunoglobulin-like receptor	2.41 ± 1.45
M14058	complement component 1, r subcomponent	2.51 ± 2.44
NM_003123	sialophorin (leukosialin, CD43)	3.01 ± 1.10
NM_000063	complement component 2	3.26 ± 1.16
NM_003319	titin	3.68 ± 1.64
NM_003804	(TNFRSF)-interacting serine-threonine kinase 1	3.69 ± 1.82
NM_004415	desmoplakin	4.91 ± 4.10
D90277	carcinoembryonic antigen-related cell adhesion	6.81 ± 4.05

### Effect of VEEV on the expression profiles of apoptosis related genes

**Figure 5:**
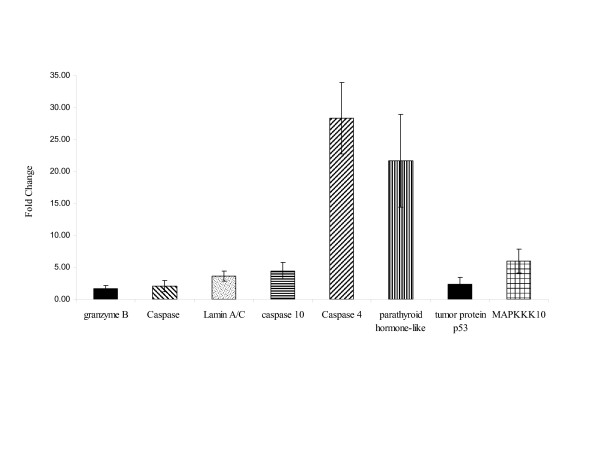
Expression of Apoptosis related genes. The caspase pathway genes, were highly up regulated in VEEV infected animals.

Ontological mining of the significantly regulated genes revealed that apoptosis related genes, and especially the caspase pathway genes, were highly up regulated in VEEV infected animals. Granzyme B, caspase 3, lamin A/C, caspase 10 and caspase 4 were all up regulated. Caspase 1 and apoptosis inhibitor 5 (API5) were down regulated in these animals when compared to the uninfected controls (Fig 5).

### Exposure to the VEEV induces the up regulation of pro-inflammatory genes

**Figure 6:**
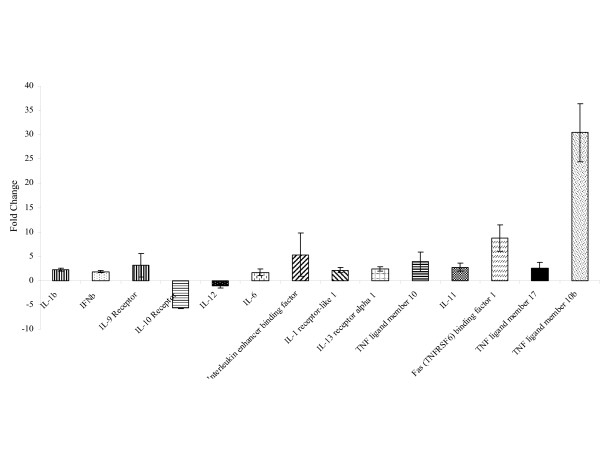
Expression patterns of pro-inflammatory genes: RNA samples were isolated and hybridized on the cDNA microarray slides as detailed in materials and methods. Images were analyzed using GenePix 4.0 and data were analyzed using GeneSpring 7.0

Exposure to the VEEV has also induced the up regulation of pro-inflammatory genes such as IL-6, IFN-b, IL-1a, IL1-b and the Fas ligand. IL-12 and IL-10 were down regulated. Figure 6 shows the expression levels of some of these genes.

### Effect of VEEV on the expression patterns of androgen related genes

**Figure 7:**
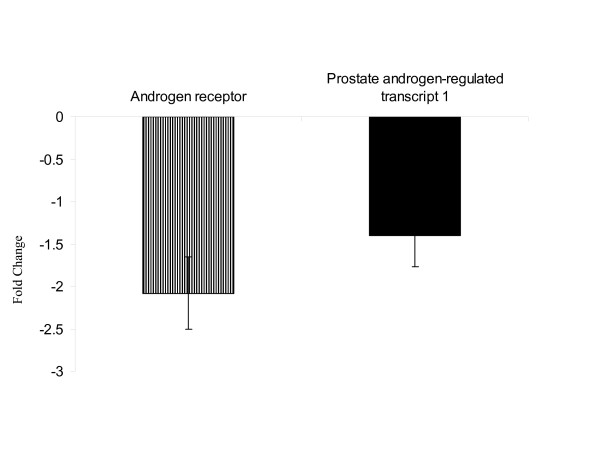
Expression patterns of androgen related genes: The androgen receptor and the Prostate androgen-regulated transcript 1 (PART1) were both down regulated by the VEEV in the blood of infected animals.

Exposure to the VEEV down regulates the expression pattern of the gene coding for the androgen receptor and the Prostate androgen-regulated transcript 1 (PART1) (Fig. 7). The androgen receptor functions as a steroid-hormone activated transcription factor [11]. Upon binding the hormone ligand, the receptor dissociates from accessory proteins, translocates into the nucleus, and then stimulates transcription of androgen responsive genes.

## Discussion

Detection of the exposure to Venezuelan equine encephalitis virus currently uses culture methods, immunoassay and gene amplification techniques. Traditional assays lack the time sensitivity that is critical in diagnosing virus infection well in advance of the onset of illness. Although new methods are improving our ability to diagnose this viral infection diagnose dramatically, they require specifically developed probes that can be circumvented by subtle sequence changes.

Recent research suggests that with sufficient knowledge of genomic expression patterns, pathogen induced changes in cellular gene expression may provide the mean to identify specific biological agents. An understanding of the internal language of the lymphocyte will also help us to understand more about the intricacies of the host-pathogen interactions and recommend potential prophylactic or therapeutic strategies.

In this study, we examined gene expression in VEEV infected NHPs using cDNA microarrays and compared the results to uninfected controls.

These animals developed fever and were viremic in response to VEEV infection. They did seroconvert in response to infection and a significant number of genes exhibited altered expression profiles that paralleled VEEV infection.

Genes related to apoptosis and the caspase pathway were significantly regulated by VEEV infection. The expression levels of Caspase 3, caspase 4, caspase 10 and lamin A/C were increased in the infected animals compared to the controls.

These alterations in gene expression may be permissive for opportunistic infections by inducing apoptosis among the affected cells.

Lower levels of expression were observed for the androgen receptor and the prostate androgen-regulated transcript 1. Muehlenbein et al had shown that the testosterone levels were down regulated upon exposure to VEEV in these animals [12]. This alteration in the androgen related genes by VEEV is suggested to be of benefit for the host by thwarting a possible testosterone-mediated immunosupression [12].

In summary, in this small sample of VEEV infected animals, expression was consistently altered in specific groups of genes that code for a wide range of biochemical functions. A few important genes of interest are discussed here.

## Conclusion

The present study, along with correlating some genes with exposure to the VEEV, identifies several novel genes as potential diagnostic and therapeutic markers for Venezuelan equine encephalitis in the blood.

## Methods

### Animals and virus

A total of 11 captive-born, adult male cynomolgus monkeys were used in this study. Research was conducted in compliance with the Animal Welfare Act and other federal statutes and regulations relating to animals and experiments involving animals and adheres to principles stated in the Guide for the Care and Use of Laboratory Animals, National Research Council, 1996. The facility where this research was conducted is fully accredited by the Association for Assessment and Accreditation of Laboratory Animal Care International. Blood samples were obtained from the monkeys on day 0 (pre-exposure). Randomly selected monkeys were exposed to a dose of 1 × 10^8 ^plaque forming units (PFU) of VEEV, the Trinidad strain, which is a virulent epizootic IA/B variant virus. At days 3, 4 and 14 post-exposure to VEEV, 2 of these monkeys were randomly selected on each day to obtain whole blood sample as described in Muehlenbein et al. [12].

### RNA isolation

Whole blood samples were collected into CPT Vacutainer tubes (BD, Franklin Lakes, NJ) and processed in accordance with the manufacturer's specifications, which allow for the enrichment of peripheral mononuclear cells (PBMC). Total RNA was subsequently isolated from PBMCs using TRIzol reagent (Invitrogen, Carlsbad, CA) following manufacturer protocol. RNA quantity was measured via spectrophotometry followed by analysis with a Bioanalyzer 2100 (Agilent Technologies, CA)

### Custom made cDNA Microarray Slide Preparation and Hybridization

Human cDNA microarrays were prepared by using sequence verified PCR elements produced from approximately 6900 well-characterized human genes of The Easy to Spot Human UniGEM V2.0 cDNA library (Incyte Genomics, Inc). The PCR products, ranging from 500 to 700 bps, were deposited in 3·saline sodium citrate (SSC) at an average concentration of 165 μg/μl on CMT-GAPS II aminopropyl silane-coated slides (Corning, Corning, NY) using a VersArray microarryer (Bio-Rad, Inc). The arrays were post processed by UV-cross linking at 1200 mJ, baked for 4 h at 80°C, and then the positively charged amine groups on the slide surface were treated with succinic anhydride/N-methyl-2-pyrrolidinone.

### Microarray hybridization and image processing

Microarray labeling was performed using Micromax Tyramide Signal Amplification (TSA) Labeling and Detection Kit (Perkin Elmer, Inc., MA). The slides were hybridized for 16 h at 60°C. The GenePix Pro 4000b (Axon Instruments, Inc., CA) optical scanner was used to scan the hybridized slides and the raw intensity was recorded through the Gene Pix 4000 software package (Axon Instruments, Inc., CA). Intensity of the scanned images was digitalized through Genepix 4.0 software.

### Microarray analysis

#### Assessment of the overall integrity of the microarray experiment

The quality of the RNA, used for microarray, was tested beforehand using a 2000 BioAnalyzer (Agilent, CA). Upon hybridization, the quality of each microarray, i.e. the efficiency of reverse transcription (RT) reactions, labeling competence etc. was assessed. Microarray images were visualized using Imagene v.6 (BioDiscovery, Inc., CA) and data were analyzed using GeneSpring V. 7.1 (Silicon Genetics, CA) and Partek Pro. V. 5.0 (Partek, MI).

#### Data cleansing and normalization

Using ImaGene (BioDiscovery Inc., CA), background and foreground pixels of each spot were segmented and the highest and lowest 2% of the probe intensity was discarded. Local background correction was applied to each individual spot. The genes that passed this filter in all given experiments were selected for further study.

Data cleansing and statistical analysis was carried out using GeneSpring^® ^7.1 (Agilent Tech., CA). Local background was subtracted from individual spot intensity. Genes that failed this 'background check' in any of the experiments were eliminated from further analysis. Each chip was next subjected to intra-chip normalization (LOWESS). The genes that varied most between control and treated sample sets were selected via *t*-test analysis.

The *p*-value cutoff was set at 0.05. Four hundreds and thirty two genes were differentially expressed between VEEV-infected and control uninfected animals with p < 0.05.

The pattern of gene expression variability of the experimental set having reduced dimension was evaluated using principal component analysis (PCA) classifying VEEV-infected and control samples as the two variable classes.

We used the reference design, where a reference RNA sample is co-hybridized with each sample on the slide. This design allows us to normalize between the slide for variations that can be due to hybridization, transcription and labeling efficiencies (technical variations).

#### Clustering

Principal component analysis (PCA) was performed over the given dataset classifying each sample as a statistical variable, in order to confirm the extent of variability within the sample classes, as well as among the pre-designed groups.

A two dimensional hierarchal clustering calculation using Pearson correlation around zero was also performed.

### Real time PCR

The t-test result was corroborated through real time polymerized chain reaction (Real-time PCR). A web-based primer designing tool was used to design the primers for the selected genes [13]. Sequences of the primers used for the selected genes are listed in table 1. The specificity of each primer sequence was further confirmed by running a blast search. Reverse transcription and Real-time PCR reactions were carried out using reverse transcription kit (Invitrogen, Carlsbad, CA) and Real-time PCR kit (Roche, IN), respectively. Each reaction with five technical duplicates was run in I-Cycler machine (Bio-Rad, CA). Each sample was also amplified using a primer set for the house-keeping probe of the experiment: glyceraldehyde 3 phosphate dehydrogenase (GAPDH). The resultant cycle threshold data from each real-time-PCR 'run' was converted to fold-change using an established algorithm [14].

**Table 1:** The sequences of the primers used in this study.

**Name**	**Gene Bank ID**	**Description**	**Sequence**	**Product Size**
COL15A	AA455157	collagen, type XV, alpha 1	5'-CCA CCT ACC GAG CAT TCT TAT C-3'	
			5'-CAA TAC GTC TCG ACC ATC AAA G-3'	197 bp
PDCD4	N71003	programmed cell death 4	5'-CCG GTG ATG AAG AAA ATG CT-3'	
			5'-TGG TTG GCA CAG TTA ATC CA-3'	207 bp
RBM9	AA451903	RNA binding motif protein 9	5'-AAC TCC TGA CTC AAT GGT TC-3'	
			5'-CAT TTT GTG TGC TGG GTG AG-3'	194 bp

Quantitative and qualitative verification of the PCR product was accomplished by running 1% agarose gel electrophoresis using SYBR Green I (Kamtek, Rockville, MD). Gel images were captured using FX Molecular Imager system (Bio-Rad, CA) scanner and analyzed using Quantity One software (Bio-Rad, CA).

## Competing interests

The author(s) declare that they have no competing interests.

## Authors' contributions

RH participated in the design of the study, carried out the microarray data analysis, data mining and participated in drafting the manuscript.

MB carried out the microarray and real time PCR studies.

GL participated in the design of the study and interpretation of the results.

SP participated in the microarray study.

NK participated in the design of the study and interpretation of the results.

MJ conceived of the study, and participated in its design and coordination. All authors read and approved the final manuscript.

## References

[B1] Johnston REPC (1995). Alphaviruses.

[B2] Vogel P, Abplanalp D, Kell W, Ibrahim MS, Downs MB, Pratt WD, Davis KJ (1996). Venezuelan equine encephalitis in BALB/c mice: kinetic analysis of central nervous system infection following aerosol or subcutaneous inoculation. Arch Pathol Lab Med.

[B3] Vogel P, Fritz DL, Kuehl K, Davis KJ, Geisbert T (1997). The agents of biological warfare. Jama.

[B4] Vogel P, Kell WM, Fritz DL, Parker MD, Schoepp RJ (2005). Early events in the pathogenesis of eastern equine encephalitis virus in mice. Am J Pathol.

[B5] (1995). Arboviral disease – United States, 1994. MMWR Morb Mortal Wkly Rep.

[B6] Weaver SC, Barrett AD (2004). Transmission cycles, host range, evolution and emergence of arboviral disease. Nat Rev Microbiol.

[B7] Calisher CH (1994). Medically important arboviruses of the United States and Canada. Clin Microbiol Rev.

[B8] http://www.babelomics.org.

[B9] Hammamieh R, Chakraborty N, Wang Y, Laing M, Liu Z, Mulligan J, Jett M (2007). GeneCite: A Stand-alone Open Source Tool for High-Throughput Literature and Pathway Mining. Omics.

[B10] http://www.biocarta.com.

[B11] Lee HJ, Mowszowicz I, Chang C (1996). The first detection of complete androgen insensitivity with no mutation in the coding sequence of the androgen receptor gene. Front Biosci.

[B12] Muehlenbein MP, Cogswell FB, James MA, Koterski J, Ludwig GV (2006). Testosterone correlates with Venezuelan equine encephalitis virus infection in macaques. Virol J.

[B13] PCR Desig. http://frodo.wi.mit.edu/cgi-bin/primer3.

[B14] Hammamieh R, Chakraborty N, Das R, Jett M (2004). Molecular impacts of antisense complementary to the liver fatty acid binding protein (FABP) mRNA in DU 145 prostate cancer cells in vitro. J Exp Ther Oncol.

